# Robotic Arm Rehabilitation in Chronic Stroke Patients With Aphasia May Promote Speech and Language Recovery (but Effect Is Not Enhanced by Supplementary tDCS)

**DOI:** 10.3389/fneur.2018.00853

**Published:** 2018-10-22

**Authors:** Adam Buchwald, Carolyn Falconer, Avrielle Rykman-Peltz, Mar Cortes, Alvaro Pascual-Leone, Gary W. Thickbroom, Hermano Igo Krebs, Felipe Fregni, Linda M. Gerber, Clara Oromendia, Johanna Chang, Bruce T. Volpe, Dylan J. Edwards

**Affiliations:** ^1^Department of Communicative Sciences and Disorders, New York University, New York, NY, United States; ^2^Restorative Neurology Clinic, Burke Neurological Institute, White Plains, NY, United States; ^3^Weill Cornell Medicine, New York City, NY, United States; ^4^Icahn School of Medicine at Mount Sinai, New York City, NY, United States; ^5^Berenson-Allen Center for Noninvasive Brain Stimulation, Beth Israel Deaconess Medical Center, Harvard Medical School, Boston, MA, United States; ^6^Fundación Institut Guttmann, Institut Universitari de Neurorehabilitació Adscrit a la UAB, Barcelona, Spain; ^7^Department of Mechanical Engineering, Massachusetts Institute of Technology, Cambridge, MA, United States; ^8^Spaulding Rehabilitation Hospital, Harvard Medical School, Cambridge, MA, United States; ^9^Center for Biomedical Science, Feinstein Institute for Medical Research, Manhasset, NY, United States; ^10^Moss Rehabilitation Research Institute, Elkins Park, PA, United States; ^11^School of Medical and Health Sciences, Edith Cowan University, Joondalup, WA, Australia

**Keywords:** aphasia, stroke rehabilitation, apraxia of speech, motor control, Neurorehabilitation, tDCS

## Abstract

**Objective:** This study aimed to determine the extent to which robotic arm rehabilitation for chronic stroke may promote recovery of speech and language function in individuals with aphasia.

**Methods:** We prospectively enrolled 17 individuals from a hemiparesis rehabilitation study pairing intensive robot assisted therapy with sham or active tDCS and evaluated their speech (*N* = 17) and language (*N* = 9) performance before and after a 12-week (36 session) treatment regimen. Performance changes were evaluated with paired *t*-tests comparing pre- and post-test measures. There was no speech therapy included in the treatment protocol.

**Results:** Overall, the individuals significantly improved on measures of motor speech production from pre-test to post-test. Of the subset who performed language testing (*N* = 9), overall aphasia severity on a standardized aphasia battery improved from pre-test baseline to post-test. Active tDCS was not associated with greater gains than sham tDCS.

**Conclusions:** This work indicates the importance of considering approaches to stroke rehabilitation across different domains of impairment, and warrants additional exploration of the possibility that robotic arm motor treatment may enhance rehabilitation for speech and language outcomes. Further investigation into the role of tDCS in the relationship of limb and speech/language rehabilitation is required, as active tDCS did not increase improvements over sham tDCS.

## Introduction

Following stroke, participation in professional, social, and family environments is often limited by acquired deficits affecting motor control, sensation, cognition, and communication. Treatment in each domain can promote some limited recovery, but residual disability remains a problem. Thus, there is a need for efficient interventions to expedite and enhance recovery. We explored the possibility that treatment in one domain—motor function—may benefit performance in another, untreated domain—communication—in individuals with acquired aphasia and/or apraxia of speech. Synergistic effects of treatment across domains could provide a transformational approach to stroke rehabilitation.

The possibility that robotic motor treatment preceded by tDCS may benefit speech-language recovery was raised by Hesse et al. (1). They reported that 4 of the 10 individuals with subacute stroke undergoing repetitive right upper-extremity robotic arm therapy in conjunction with transcranial direct current stimulation (tDCS) exhibited unexpected improvement on a standardized speech-language measure. The present investigation extended this examination to the chronic stage of recovery, using tDCS in conjunction with a 12-week course of right upper-extremity robotic arm therapy. To determine whether changes in the untreated speech-language domain would occur in this population, eligible individuals with chronic stroke (>6 months post onset) were prospectively enrolled. These participants were a subset of a larger randomized control trial of hemiparesis recovery using intensive robotic arm treatment preceded by tDCS.

## Methods

### Participants

A subset of seventeen participants (10 M, 7 F) from a large multi-site RCT were enrolled in the speech and language study reported here (dates: 4/2013-9/2014). All treatment and assessments were conducted at Burke Medical Research Institute and Feinstein Institute for Medical Research with participants consented under IRB approval of both institutions. Eligibility criteria for the larger RCT were: a single, left-hemisphere ischemic lesion; cognitive function sufficient to understand the instructions and task in the study; and a Motor Power score ranging from 1 to 4/5, indicating that participants were neither hemiplegic nor had fully recovered motor function in the muscles of the shoulder, elbow, and/or wrist. Participants in the speech and language subset also presented with chronic aphasia and/or apraxia of speech subsequent to the same lesion. Speech-language testing occurred prior to the beginning of motor therapy (pre-test baseline) and again after the conclusion of the therapy sessions (post-test). All 17 speech-language participants were administered a speech motor control task (i.e., diadochokinetic rate). Language testing was only conducted on individuals whose primary language was English (*N* = 11) because the normative samples for the measures given are based on native English speakers. Two English speakers were unable to complete the full battery at post-test, leaving complete language data sets for 9 people for the Western Aphasia Battery-R and 10 people for the category naming task. Speech motor control data for 17 participants is reported here. All participants completed the entire 36 session hemiparesis protocol.

### Testing battery

#### Diadochokinesis

Diadochokinetic rate (DDK) assesses the ability to produce sequential and alternating syllables (e.g., “papapa;” “pataka”). We used the test version from the Apraxia Battery for Adults 2nd version (ABA-2) (2) and followed the scoring protocol outlined in the examiner's manual.

#### Category naming

Participants were given one minute to name members of a semantic category (animals; transportation; plants; tools). This frequently used verbal fluency measure requires lexical access and word retrieval (3). Unimpaired speakers typically produce 20+ category members per minute.

#### Comprehensive speech-language battery

The Western Aphasia Battery-Revised (WAB-R) (4) is a comprehensive aphasia battery, and yields an overall “aphasia quotient” (AQ) indicating aphasia severity (0–100). Scores above 93.8 are within the normal range; changes of 5+ points are considered clinically significant (5).

### Procedure

All participants were enrolled in the double-blind repetitive right upper-extremity motor therapy study that included 36 sessions (3x/week) of robotic arm motor therapy (~1 h each) preceded by 20 min of tDCS. In this study, a 2 mA current was delivered by a battery-driven current generator using surface rubber-carbon electrodes (5 × 7 cm; 35 cm^2^) surrounded by saline-soaked sponges. The center of the anode was placed 5 cm lateral to the vertex, and the cathode was placed over the right supraorbital region. For participants in the sham tDCS condition, current was ramped up to 2 mA over 30 s and then ramped down to 0 ms (6). The Fugl-Meyer Assessment (FM) (7) scores were obtained pre-test, post-test, and at a 6-month follow-up (not reported here). The FM provided a standardized assessment of upper extremity abilities administered by a licensed occupational therapist or trained research assistant.

Speech-language tasks were administered by an ASHA-certified speech-language pathologist (SLP). Participants were assessed during the week prior to the first treatment session (pre-test baseline), and within 5 days of the last treatment session (post-test). The same SLP administered baseline and post-test evaluations for each participant. Participants did not receive speech-language therapy as part of the treatment.

### Data analysis

To evaluate changes, we performed two analyses for each speech-language measure: a within-subjects paired *t*-test to evaluate overall change from baseline to post-test; and an independent samples *t*-test using the difference scores for each participant to evaluate tDCS (active~sham) group differences. Despite the small sample size, we use *t*-tests to allow for confidence intervals to get a sense of the effect size, and we also report the outcomes of non-parametric Wilcoxon tests throughout the results.

## Results

Demographic and stroke information, as well as tests they completed and tDCS group assignment, are presented in Table 1. Consistent with the larger data set and prior published studies, Fugl-Meyer Assessment (FM) (7) scores among these patients significantly increased following the robotic training regimen (mean: 27.3 pre, 35.4 post). Mean pre-to-post increase was 8.1 points [95% confidence interval (CI; 6.4, 9.8); *t*-test: *p* < 0.001; Non parametric Wilcoxon: *p* < 0.001]. As in the full cohort from the robotic arm study, improvement did not differ between tDCS and sham arms in the full set of 17 participants (*t*-test: *p* = 0.14; Non parametric Wilcoxon: *p* = 0.13) or in the subset with WAB data discussed below (*t*-test: *p* = 0.28; Non parametric Wilcoxon: *p* = 0.34).

**Table 1:** Participant information: age at onset of study (within 5 year range), number of months post-stroke, handedness, race, damage type (cortical/subcortical/mixed), tDCS condition, and which speech/language measures were completed.

**Sub #**	**Age (5 year range)**	**Post CVA (mos.)**	**Hand**	**Race**	**Damage**	**tDCS**	**DDK**	**Category naming**	**WAB**	**DDK severity**	**Aphasia type**
S1	70–75	48	RH	Caucasian	Cortical	Sham	Y	Y	Y	Severe	Transcortical motor
S2	65–70	112	RH	Caucasian	Cortical	Active	Y	Y	Y	Moderate	Anomic
S3	45–50	72	RH	Caucasian	Mixed	Sham	Y	Y	Y	Mild	Broca's
S4	70–75	36	RH	Asian	Cortical	Sham	Y	Y	Y	Mild	Anomic
S5	60–65	8	RH	Caucasian	Mixed	Sham	Y	Y	Y	Mild	Anomic
S6	75–80	12	RH	Caucasian	Mixed	Active	Y	Y	Y	None	Anomic
S7	60–65	48	RH	Caucasian	Cortical	Active	Y	Y	Y	Mild	Conduction
S8	60–65	24	RH	African-American	Mixed	Sham	Y	Y	Y	Mild	Broca's
S9	45–50	228	RH	Caucasian	Subcortical	Active	Y	Y	Y	None	Anomic
S10	45–50	45	RH	Caucasian	Cortical	Active	Y	Y	N	Moderate	n/a
S11	60–65	104	RH	Caucasian	Cortical	Active	Y	N	N	Mild	n/a
S12	75–80	56	LH	Caucasian	Subcortical	Active	Y	N	N	Mild	n/a
S13	75–80	11	RH	African-American	Subcortical	Sham	Y	N	N	None	n/a
S14	75–80	47	RH	Caucasian	Subcortical	Sham	Y	N	N	Mild	n/a
S15	80–85	6	RH	Caucasian	Cortical	Sham	Y	N	N	Mild	n/a
S16	70–75	42	RH	Caucasian	Mixed	Active	Y	N	N	Mild	n/a
S17	45–50	26	RH	Caucasian	Mixed	Active	N	Y	N	n/a	n/a

*Diadochokinetic (DDK) severity ratings come from subtest 1 of the Apraxia Battery for Adults, 2nd edition (ABA-2)^2^ and aphasia diagnoses come from the Revised Western Aphasia Battery (WAB-R)^4^ at pre-test baseline*.

### Diadochokinetic (DDK) task

**Figure 1:**
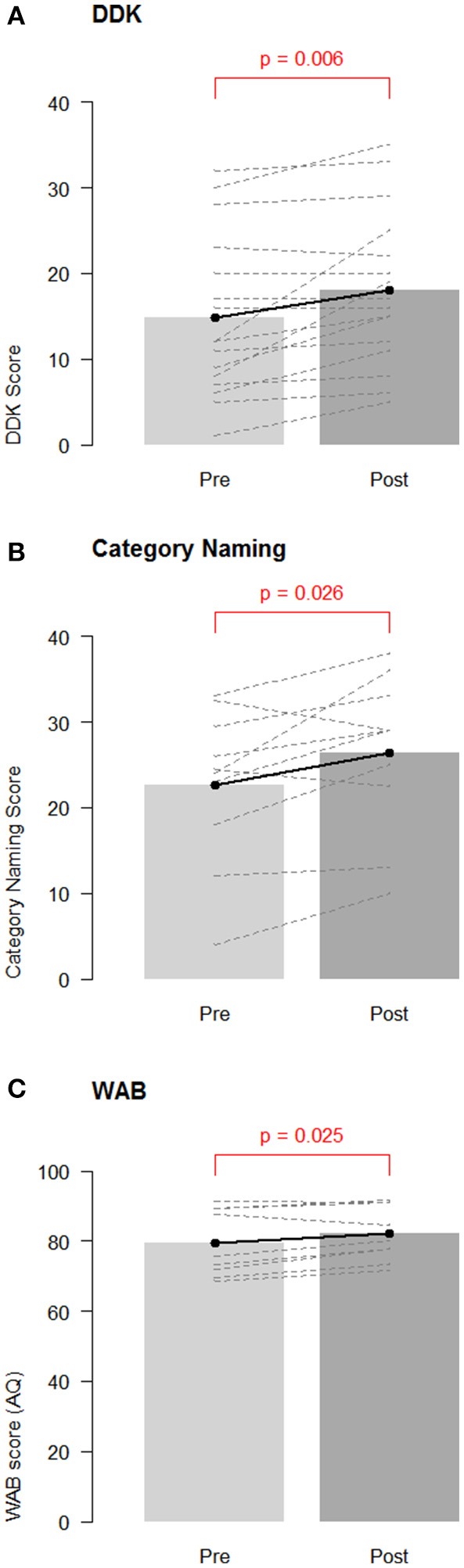
Group changes in performance on speech/language measures. Overall pre-test baseline and post-test changes for **(A)** diadochokinetic rate; **(B)** category naming score; and **(C)** Western Aphasia Battery—Aphasia Quotient (WAB AQ). Barbells represent overall means, and dotted lines represent individual participants.

A paired *t*-test revealed significant improvement in DDK scores from baseline to post-test [mean: 3.19 points, CI (1.04, 5.34); *t*-test: *p* = 0.006, Non parametric Wilcoxon *p* = 0.003; Figure 1A]. There was no difference between participants receiving sham (*N* = 8) and active tDCS [*N* = 8; difference = −0.38, CI (−4.10, 4.85), *t*-test: *p* = 0.86].

### Category naming

A paired *t*-test revealed a significant increase in category naming scores from baseline to post-test overall [mean: 3.27 items, CI (0.16, 6.39), *t*-test: *p* = 0.026, Non parametric Wilcoxon *p* = 0.036; Figure 1B]. Participants in the sham group (*N* = 5; mean improvement: 6.60 items) improved by significantly more items than participants in the active tDCS group [*N* = 6; mean improvement: 0.50 items; estimated difference = 6.10, CI (1.28, 10.92), *t*-test: *p* = 0.041, Non parametric Wilcoxon = 0.075].

### Comprehensive speech-language battery

A paired *t*-test revealed significant improvement in the WAB-R AQ from baseline to post-test [mean: 2.51 points, CI (0.41, 4.61), *t*-test *p* = 0.025, Non parametric Wilcoxon = 0.055, see Figure 1C]. This indicates that the participants improved overall in their average WAB-R performance at post-test. One participant demonstrated a clinically significant improvement of at least 5 points (sham condition) and two other participants achieved improvements of 4.6 and 4.9 points (one active and one sham). No significant group differences were seen between participants receiving active (*N* = 4) and sham tDCS [*N* = 5; mean difference: −3.08, CI (−7.90, 1.74), *t*-test *p* = 0.15; Non parametric Wilcoxon *p* = 0.19].

## Discussion

In our data, chronic stroke participants with speech and/or language impairment exhibited detectable improvement on speech-language measures following intensive robotic arm rehabilitation preceded by tDCS. Critically, this improvement was observed in the absence of speech-language therapy, suggesting the possibility of synergistic effects across these distinct domains of stroke recovery. It is worth noting that Meinzer et al. (8) reported that tDCS stimulation with this same montage may provide benefits to language processing in older neurotypical adults, suggesting the possibility that gains in language ability could come from the stimulation. However, in our limited data set, there was no effect of tDCS condition in most tasks. While we must be cautious about drawing strong conclusions from this dataset, it is possible that this reflects the fact that there was no explicit speech/language activity paired with the stimulation and that any benefit tDCS may provide in stroke rehabilitation comes from pairing of treatment and stimulation. We also note that recent findings on whether tDCS can affect language processing have been mixed, with recent positive findings reported for tDCS as an adjunct to aphasia therapy (9) and stuttering therapy (10) but null findings also widely reported (11).

In addition, we note that participants receiving sham showed a greater improvement on category naming than those receiving active stimulation, with the difference reaching significance in a *t*-test (but not a non-parametric test). This result leaves open the possibility that active tDCS was actively detrimental to improvement on this task, although it is surprising that this finding would occur for only one measure. We also note that tDCS groups were not matched for speech-language ability as part of the RCT and they were not matched in the study. The small sample size here also precludes us from determining whether other demographic or neurological factors (such as those outlined in Table 1) can account for this difference, as any regression analysis that would address this would be problematic due to the number of participants.

The positive overall findings should be treated cautiously in the absence of a control group not receiving robotics. In addition, the finding of an improvement on the WAB was only significant for a *t*-test and not non-parametric tests, and the 95% confidence intervals for that comparison fall below the 5 point threshold used to identify clinically significant improvement on that test. Nevertheless, these findings, in addition to those in Hesse et al. (1) on the subacute population, warrant additional systematic explorations of the benefit of multi-domain therapies for stroke rehabilitation.

## Author contributions

AB and CF contributed equally to this manuscript. AB contributed to study concept and design, data analysis and interpretation, drafted, and revised manuscript for intellectual content. CF and DE contributed to study concept and design, data analysis and interpretation, critical revision of manuscript for intellectual content. AR-P contributed to study design. MC, AP-L, GT, HK, FF, JC, and BV contributed to study concept and design. LG and CO contributed to data analysis and interpretation, and critical revision of manuscript for intellectual content.

### Conflict of interest statement

AP-L serves on the scientific advisory boards for Nexstim, Neuronix, Starlab Neuroscience, Neuroelectrics, Axilum Robotics, Magstim Inc., and Neosync; and is on several issued and pending patents on the real-time integration of transcranial magnetic stimulation (TMS) with electroencephalography (EEG) and magnetic resonance imaging (MRI). The remaining authors declare that the research was conducted in the absence of any commercial or financial relationships that could be construed as a potential conflict of interest.
